# International Multicenter Validation of an Expanded AI Diagnostic System for 18 Pathologies in Thoracic and Musculoskeletal Radiography

**DOI:** 10.3390/diagnostics16081137

**Published:** 2026-04-10

**Authors:** Jean-Laurent Sultan, Pauline Beaumel, Maria Dementjeva, Hugo Declercq, Ilana Sultan, Julia Reinas, Maria Dolores Durán Vila

**Affiliations:** 1 Groupe Alloradio (Simago) Private Imaging Medical Center, Montalembert, 9 Rue de Montalembert, 75007 Paris, France; 2 AZmed, 10 Rue d’Uzès, 75002 Paris, France; 3 SA Narva Haigla (Narva Hospital), Haigla 1, 20104 Narva, Estonia; 4 Department of Radiology, az Sint-Blasius, Kroonveldlaan 50, 9200 Dendermonde, Belgium; 5 Servicio de Radiodiagnóstico, Complejo Hospitalario Universitario de Pontevedra (CHUP), Calle Mourente s/n, 36071 Pontevedra, Spain

**Keywords:** artificial intelligence, deep learning, computer-aided diagnosis, fracture detection, musculoskeletal radiology, bone lesion detection, thoracic imaging, chest abnormalities, Rayvolve^®^ AI suite

## Abstract

**Background:** Conventional radiography faces high error rates (3–10%) due to heavy clinical workloads. While AI has emerged as a supportive tool, there is an evidence gap regarding the clinical utility of integrated AI systems in detecting both skeletal and thoracic abnormalities. **Objectives:** This large-scale, international multicenter study aims to validate the performance of a unified radiographic AI suite across an expanded diagnostic scope while confirming its continued robustness. **Methods:** A retrospective performance evaluation was conducted using 21,581 adult and pediatric X-rays collected from 20 countries. The reference standard was established through independent review by two expert readers, with adjudication of a third radiologist in cases of discordance. Diagnostic metrics, including Area Under the Curve (AUC), sensitivity, and specificity, were calculated for all 18 pathologies. Subgroup analysis was performed by patients’ age, sex, and country of acquisition. **Results:** For the nine findings within the expanded scope, AUC values exceeded 96.1%, with sensitivity and specificity ranges from 94.5 to 98.8% and 86.6 to 96.1%, respectively. Similarly, for the nine historically validated findings, AUCs remained above 96.1%, with sensitivity and specificity localized between 94.5 and 97.8% and 84.6 and 89.4%, respectively. Consistency was maintained across subgroups. **Conclusions:** The results confirm the potential of deep learning to transition from narrow, task-specific tools to a unified, high-performance diagnostic system.

## 1. Introduction

Conventional radiography remains the main diagnostic tool used in clinical practice, representing approximately 60% to 70% of all radiological exams [1,2,3]. Despite its essential role, the large volume of X-ray interpretation, further strained by a global shortage of radiologists, raises the risk of diagnostic errors and professional burnout [4,5,6]. Error rates for detecting acute findings are estimated to range between 3% and 10%, often due to the increased cognitive burden and the subjective nature of manual interpretation in daily practice [4,5].

Over the last decade, artificial intelligence (AI) has emerged as a robust adjunct for specific, high-frequency tasks, transitioning from early knowledge-based systems to advanced deep learning (DL) architectures [7,8,9]. Modern DL models, especially multi-layered convolutional neural networks (CNNs), are highly effective at automatically learning complex features from large and diverse datasets, with performance improving as they are trained on more varied data over time [10,11,12]. However, a significant limitation in current AI research is its focus on narrow, single-domain applications, often confined to specific areas such as detecting appendicular fractures or analyzing limited chest abnormalities [7,13,14].

This fragmentation poses a practical challenge in clinical settings, where the increasing use of specialized point solutions for specific tasks can lead to fragmented workflows and reduced efficiency. There is a notable lack of clinical research evaluating unified AI systems designed to diagnose both whole-body skeletal pathologies and thoracic conditions simultaneously within a single, integrated workflow [15]. Most existing research focuses on these areas separately, without evaluating the clinical usefulness of an integrated system capable of characterizing a comprehensive spectrum of pathology across different body regions [16,17]. While specific components of the Rayvolve^®^ AI suite (AZmed, Paris, France) have been previously validated in the literature for specific tasks [18,19,20,21], a significant gap remains in evidence regarding its effectiveness in analyzing other complex or high-volume radiological signs within a unified diagnostic framework [22,23,24].

The need for such an integrated approach is further underscored by the high mortality and morbidity associated with missed findings in chronic and infectious diseases, such as tuberculosis, which often exhibit diverse radiographic phenotypes that current narrow-scope AI models do not fully address [25,26,27].

The present study describes a large-scale, international, multicenter, retrospective performance evaluation of a unified radiographic AI suite with an expanded diagnostic scope. The primary objective is to validate the system’s performance across a broader clinical range that has not been collectively addressed in the literature, including hilar/mediastinal adenopathy, lung cavities, tuberculosis, focal bone lesions, interstitial patterns, mediastinal widening, pneumonia, atelectasis, and old fractures. The secondary objective is to confirm its ongoing robustness in diagnosing well-established findings such as cardiomegaly, consolidation, pneumothorax, pulmonary nodules, pleural effusion, pulmonary edema, acute/subacute fractures, dislocations, and joint effusions [18,19,20,21]. By exploring this wider diagnostic range, the study aims to set a new standard for comprehensive, multi-domain AI-assisted radiographic interpretation [28].

## 2. Materials and Methods

This study was conducted and reported to the furthest extent possible, according to the Standards for Reporting Diagnostic Accuracy AI (STARD-AI) guideline [29]. However, the scope and context of our study introduced specific limitations. Consequently, some specific reporting items, including the participant flow diagram, were not applicable or feasible due to the constraints imposed by the data structure.

### 2.1. Study Design

This research was designed as a retrospective, multicenter, international diagnostic performance study. A total of 21,581 X-rays were collected between September 2021 and December 2025 from 52 medical centers across 20 countries spanning five continents. The cohort represents a diverse range of clinical environments, from high-volume tertiary academic hospitals to specialized outpatient imaging centers, thereby enhancing the external validity of our findings.

The study was conducted in accordance with the ethical principles of the Declaration of Helsinki (revised in 2013). All data involved were secondary-use, retrospective radiographs that were fully anonymized at the source facilities prior to their transfer to the central analysis site. The anonymization process was performed by removing all Protected Health Information and metadata potentially identifying the patient, in compliance with the ISO 25237:2017 standard [30] for health informatics pseudonymization and anonymization.

Given the retrospective nature of the study and the use of non-identifiable data, the requirement for individual informed consent was waived at all participating sites. The study was categorized as non-human subject research as defined by 45 CFR 46 in the United States along with corresponding international regulations. Data acquisition was conducted in compliance with the MR-004 reference methodology established by the French National Commission on Informatics and Liberty (CNIL). This framework governs the processing of personal health data utilized for research purposes, specifically in scenarios involving anonymized data.

### 2.2. Inclusion and Exclusion Criteria

#### 2.2.1. Inclusion Criteria

To ensure the study reflected the full complexity of daily clinical practice, broad and inclusive criteria were applied. Digital radiographs were included if they met the following requirements:

• Examinations acquired via direct digital radiography (DR) or computed radiography (CR) systems, including bedside imaging performed with portable units. Radiographic fluoroscopy (RF) images from musculoskeletal (MSK) examinations were also included if they were in DICOM format.
• Thoracic Imaging: All chest X-rays from inpatient and outpatient settings, including Anteroposterior (AP), Posteroanterior (PA), and lateral views. All patient positions were included, meaning X-rays acquired in standing, sitting, and supine positions, as well as lateral decubitus views when available.
• MSK Imaging: The entire appendicular and axial skeleton, including upper and lower limbs, pelvis, and the spine (cervical, thoracic, lumbar), encompassing all standard radiographic projections, weight-bearing (under load) and non-weight-bearing positions.
• Patient Demographics: There was no age restriction. The study intentionally included the full pediatric spectrum, including neonates and infants under 1 year of age, as well as adult and geriatric populations. The Rayvolve^®^ AI suite is designed and CE-marked for use across all age groups without restriction. Its performance in pediatric populations, characterized by complex skeletal maturation and open growth plates, has been specifically validated in previous multicenter studies [23,26], while its thoracic capabilities have been established across broad clinical cohorts [25].

#### 2.2.2. Exclusion Criteria and “Real-World” Quality Paradigm

The exclusion criteria were strictly limited to maintain the focus on the system’s supported diagnostic horizon:

• Anatomical Exclusions: Radiographs of the skull, facial bones, and dental imaging were excluded as these regions are not currently supported by the Rayvolve^®^ AI Suite.
• No Technical Exclusions: No images were excluded based on technical quality. To evaluate the system’s performance within a natural distribution of clinical data, radiographs with suboptimal exposure (over/under-exposed), patient rotation, or overlapping medical devices (e.g., tubes, lines, or implants) were retained in the final cohort. This approach was adopted to avoid “cherry-picking” bias and to rigorously assess the AI’s robustness against the inherent heterogeneity of global radiological workflows.

#### 2.2.3. Metadata and Metadata Extraction

Each examination was associated with localized metadata, including the country of acquisition, patient age, and sex. No standardization of imaging protocols (e.g., kiloVoltage (kVp) or milliampere-seconds (mAs)) was imposed, allowing for the inclusion of varied image resolutions and exposure parameters from a wide array of equipment manufacturers across the 52 participating centers.

### 2.3. AI System Description and Inference

The system evaluated was the Rayvolve^®^ AI suite version 7.0.0 (AZmed, Paris, France, https://www.azmed.co/). The system utilizes an ensemble algorithm composed of multiple object detection models based on the RetinaNet framework [31]. To optimize feature extraction across varying image qualities, the current iteration of the suite employs an advanced dual-backbone architecture comprising High-Resolution Net (HRNet) and Pyramid Vision Transformer (PVT) [11].

A comparative analysis against previously employed models, such as Visual Geometry Group (VGG) -16 [10] and DenseNet, highlights a critical shift from traditional CNNs to modern, hybrid frameworks. Legacy CNNs rely on progressive downsampling, which effectively aggregates features but inherently sacrifices the high-resolution spatial details needed to detect subtle radiographic abnormalities, such as hairline fractures and small pulmonary nodules. Architecturally, HRNet addresses this limitation by maintaining continuous, parallel, high-resolution representations throughout the network for precise spatial localization. Furthermore, whereas older models are constrained by localized receptive fields, the integration of PVT introduces multi-scale self-attention mechanisms. This allows the model to capture long-range global anatomical dependencies, resulting in a highly effective combined approach for both detailed local feature extraction and comprehensive global image understanding.

The object-detection architecture comprised trainable convolutional layers, non-trainable max-pooling layers, and trainable batch-normalization layers. Each sub-model generated a set of bounding boxes, characterized by pathology classifications and associated confidence scores. To synthesize the final output, a five-model ensemble strategy was employed based on a majority voting mechanism.

Specifically, a bounding box was retained only if it demonstrated a spatial overlap across at least three independent models for the same pathology. Among overlapping proposals, the bounding box with the highest confidence score was selected, ensuring each model contributed equally to the consensus (unweighted voting). As a final post-processing step, pathology-specific thresholds were applied to filter predictions. These thresholds were optimized using an independent validation dataset, strictly disjoint from the training set, to ensure unbiased performance.

The Rayvolve^®^ AI suite is able to detect clinical pathologies (such as Pneumonia, Tuberculosis, Pulmonary Edema, Atelectasis, Fractures, and Dislocations) and radiological signs or morphological findings (which include Pleural Effusion, Consolidation, Mediastinal Widening, Interstitial patterns, Adenopathy, Lung Cavities, Joint Effusion, Cardiomegaly, Pulmonary Nodules, and Focal Bone Lesions).

The ensemble automatically identifies and applies the relevant algorithm among two specialized verticals:

*1. AZtrauma*: Detects and localizes old and acute/subacute fractures, dislocations, joint effusions [32], as well as focal bone lesions. The system is specifically engineered to discriminate between different temporal stages of bone healing based on distinct radiographic features. Acute fractures are characterized by sharp, well-defined cortical breaks and a lack of reparative bone. Subacute fractures (typically 1 to 4 weeks old) exhibit the early signs of biological repair, such as a subtle blurring of fracture margins and the initial appearance of periosteal reaction or soft callus formation. Old fractures represent established sequelae, characterized by consolidated bony callus, cortical remodeling, and the rounding or sclerosis of the fracture edges, indicating a completed or near-completed healing process. To enhance feature extraction and accurately capture subtle morphological variations, the deep learning models were trained using a granular three-class labeling architecture (acute, subacute, and old). However, to ensure clinical relevance and synchronize with emergency protocols, where the primary objective is the identification of recent injuries, the system ultimately condenses these findings into a binary classification. This yields two categories: acute/subacute, which indicates findings that may necessitate clinical intervention, and old, signifying chronic conditions or radiological sequelae.

*2. AZchest*: Detects and localizes thoracic abnormalities, including cardiomegaly, consolidation, pneumothorax, nodules, pleural effusions, pulmonary edema [33], as well as interstitial patterns, mediastinal widening, hilar/mediastinal adenopathy, lung cavities, tuberculosis, atelectasis, and pneumonia. It has a multi-layered diagnostic logic that distinguishes between elementary radiological signs and syndromic interpretations. The system employs a hierarchical approach to tuberculosis detection. It is trained to identify specific radiological markers highly suggestive of the disease, such as lung cavities, hilar/mediastinal adenopathy, and interstitial patterns. Crucially, while the algorithm utilizes the co-occurrence of these features to infer a high-probability “tuberculosis” label, it is also capable of detecting each sign independently. This ensures that the system can identify non-tuberculous pathologies while maintaining a high sensitivity for tuberculosis screening. The algorithm identifies “Consolidation” as a primary radiological sign. Given that consolidation is a non-specific finding, the system is designed to differentiate its clinical implications: it can infer a “Pneumonia” label when patterns are suggestive of an infectious process, but it also considers other differentials, such as atelectasis or focal edema. This dual-pathway allows the AI to act both as a detection tool for focal opacities and as a diagnostic aid for infectious syndromes. The suite is applicable to adult and pediatric x-rays, and has been validated in several peer-reviewed publications assessing its performance in both adult and pediatric populations [18,19,20,21]. For each analysis, the algorithm generates a three-level classification output (presence, doubt, or absence) and produces a duplicate radiograph with bounding boxes highlighting the detected findings.

### 2.4. Reference Standard Determination

To establish a high-quality Ground Truth (GT), a rigorous multi-step annotation process was implemented, following the “independent double-reading with adjudication” paradigm.

#### 2.4.1. Expert Panel and Task Distribution

The panel consisted of ten expert readers: radiologists (>5 years of experience) and senior specialized radiographers (>10 years of experience). To maximize diagnostic accuracy, readers were assigned cases strictly within their area of expertise: thoracic specialists reviewed chest radiographs, while MSK specialists reviewed appendicular and axial skeletal examinations. Crucially, the annotation scope for radiographers was strictly restricted to MSK and bone-related findings, leveraging their specialized clinical expertise in trauma radiography. All thoracic imaging and complex chest pathologies were exclusively evaluated by board-certified radiologists. Prior to the independent reading phase, all panel members underwent a standardized calibration session. This included familiarization with the annotation software and alignment on the specific operational definitions for each radiological finding to ensure uniform diagnostic criteria across the panel.

#### 2.4.2. Annotation Methodology

Reviewing was performed via a dedicated, secure web-based annotation platform equipped with standard radiological tools (zoom, contrast, and brightness adjustment).

Each radiograph was independently reviewed by two readers. For the purpose of this study, the GT was established at the case level. Agreement was defined as both readers identifying the same radiological finding within the same examination.

In cases of discordance regarding the presence, absence, or nature of a finding, a third radiologist acted as an adjudicator to provide the final decision. The initial discordance rate was 4.74%, consistent with inherent inter-observer variability in radiological practice [34].

#### 2.4.3. Blinding and Mitigation of Bias

The labeling phase was conducted under strict blinding protocols to prevent diagnostic bias:

• Readers had no access to the AI suite’s predictions, bounding boxes, or confidence scores.
• All readers were blinded to original clinical indications, patient history, referral notes, and previous radiological reports.
• No access was provided to collateral imaging (e.g., CT or MRI) or longitudinal follow-up data.
• Inter-reader Blinding: Each of the first two readers was blinded to the other’s findings during the initial independent phase.

### 2.5. Data Analysis Plan and Statistical Analysis

For each finding, the AI prediction was compared to the GT at the case level. Because the system performs multi-label classification, multiple co-existing pathologies within the same radiograph were evaluated independently for each class. A case was classified as a True Positive (TP) if the AI identified the same finding as the GT within the examination. Conversely, a False Positive (FP) occurred if the AI predicted a finding absent in the GT, while a True Negative (TN) was recorded when both the AI and GT identified no findings. A False Negative (FN) was defined as a GT finding not detected by the AI. The Jaccard distance (also called the Intersection-over-Union (IoU)) between each GT bounding box and each predicted bounding box was used in order to compute the positives and negatives findings was 25%. This implies that a predicted bounding box was classified as a True Positive only if the area of overlap between the AI’s detection and the GT accounted for at least one-quarter of their total combined area (union).

The primary endpoint was the Area Under the Receiver Operating Characteristic Curve (AUC ROC). The secondary endpoints comprised fundamental diagnostic metrics, including sensitivity, specificity, positive predictive value (PPV), and negative predictive value (NPV).

The findings were categorized into two distinct objectives for analysis. The primary objective (Expanded Diagnostic Scope) was to validate the performance of the system across a broadened clinical horizon, including hilar/mediastinal adenopathy, lung cavity, tuberculosis, focal bone lesion, interstitial pattern, mediastinal widening, pneumonia, atelectasis, and old fracture.

Secondary objective (Historically Validated Scope) was to confirm continued diagnostic robustness on standard findings previously established in clinical literature, including cardiomegaly, consolidation, pneumothorax, pulmonary nodule, pleural effusion, pulmonary edema, acute/subacute fracture, dislocation, and joint effusions.

To prioritize the system’s utility as a clinical safety net, any result classified by the algorithm as “doubt” was binarized as a positive finding. Because this three-tier output (absence/doubt/presence) is an optional feature not uniformly enabled across all participating clinical centers, any finding reaching at least this doubt threshold was binarized as a positive result to homogenize the multicenter data for statistical analysis. While this binarization inherently accepts a marginal increase in false positive rate, thereby negatively impacting specificity, it deliberately prioritizes sensitivity to ensure the system functions as a rigorous clinical safety net where no potential acute pathology is overlooked during initial triage. Confidence intervals (95% CI) for AUC were derived using bootstrap resampling with 2000 samples, whereas 95% CIs for sensitivity, specificity, PPV, and NPV were determined using the Wilson score method. The AUC ROC was derived using the continuous confidence scores produced by the ensemble algorithm. Specifically, for each of the eighteen findings, the highest probability score assigned to a detection within the examination was utilized as the continuous variable for the ROC analysis. This approach allows for a rigorous evaluation of the model’s performance across all possible operating thresholds, independent of the discrete ordinal categories provided in the clinical interface.

Subgroup analyses were stratified by sex, age, country of acquisition, and body region for MSK X-rays. The evaluation cohort was strictly sequestered and remained entirely independent from the developmental datasets employed during the training of the Rayvolve^®^ AI suite.

## 3. Results

### 3.1. Dataset Characteristics

A total of 21,581 radiographs met the inclusion criteria and were successfully processed by the AI suite. The final evaluation cohort was characterized by a balanced gender distribution (49.9% male vs. 50.1% female) and a significant pediatric representation (*n* = 5309; 24.6%).

The dataset was nearly equally partitioned between the two specialized algorithms: the AZtrauma vertical accounted for 11,125 x-rays across seventeen different body regions, while the AZchest vertical encompassed 10,456 thoracic x-rays.

Geographically, the data originated from 52 international sites spanning 20 countries across 5 continents, with the highest contributions from Spain (11.4%), the United States (10.7%), and the United Kingdom (9.6%). No radiographs were excluded due to processing errors or technical incompatibility, resulting in a 100% success rate for automated inference.

All the dataset characteristics are detailed in Table 1.

### 3.2. Primary Objective: Performance Across the Expanded Diagnostic Scope

The primary objective evaluation demonstrated high discriminative capacity across the expanded diagnostic spectrum. For the findings included in this expanded clinical field, the algorithm achieved the AUC values consistently above 96.1%.

Across these pathologies, diagnostic performance remained robust, with sensitivity and specificity values consistently localized between 94.5% [95% CI: 92.8–95.8%]–98.9% [95% CI: 96.8–99.6%] and 86.6% [95% CI: 85.9–87.2%]–96.1% [95% CI: 95.8–96.5%], respectively.

The highest diagnostic accuracy was observed for focal bone lesions, yielding a near-perfect AUC of 99.2% [95% CI: 0.9845–0.9970] and the highest specificity of the series (96.1% [95% CI: 95.8–96.5%]). Conversely, while remaining within a high-performance range, the lower bound of the diagnostic spectrum was observed for atelectasis and old fractures, with AUCs of 96.1% and 96.2%, respectively. Specifically, old fractures exhibited the lowest sensitivity (94.5% [95% CI: 92.8–95.8%]).

Detailed performance metrics for the primary objective are summarized in Table 2.

### 3.3. Secondary Objective: Performance Across Historically Validated Scopes

The secondary objective analysis confirmed stable performance for findings previously established in the clinical literature.

For thoracic x-rays, findings including cardiomegaly, consolidation, pneumothorax, pulmonary nodules, pleural effusions, and pulmonary edema demonstrated AUC values consistently above 96.1% [95% CI: 94.7–97.4%]. For these thoracic abnormalities, sensitivity and specificity ranges were maintained between 94.5% [95% CI: 91.0–96.7%] and 97.8% [95% CI: 96.5–98.6%] and 84.6% [95% CI: 83.9–85.3%] and 89.4% [95% CI: 88.8–90.0%], respectively.

For MSK X-rays, the system achieved an AUC exceeding 97.0% [95% CI: 95.9–97.9%] for acute/subacute fractures, dislocations, and joint effusions. Correspondingly, sensitivity and specificity for these traumatic musculoskeletal findings were localized between 95.8% [95% CI: 93.6–97.2%] and 97.8% [95% CI: 96.4–98.7%] and 85.2% [95% CI: 84.5–85.9%] and 86.8% [95% CI: 86.1–87.5%], respectively.

Analysis of individual findings reveals that joint effusion and cardiomegaly were the top performers, with AUC values of 98.0% and 97.9%, respectively. Conversely, the lower end of the high-performance spectrum included consolidation and pulmonary nodules, both yielding an AUC of 96.1%. Additionally, pneumothorax exhibited the lowest relative sensitivity (94.5% [95% CI: 91.0–96.7%]).

Detailed performance metrics for the secondary objective are summarized in Table 3.

### 3.4. Subgroup Performance and Generalizability

The global performances of AZtrauma and AZchest were computed as the mean of all the findings included in both verticals (with both extended and historical scopes).

Subgroup analyses confirmed the generalizability of the Rayvolve^®^ AI suite. Diagnostic consistency was maintained across all demographic and geographic variables. For both AZtrauma and AZchest, the AUC remained stable across all subgroups, consistently exceeding 94.4% [95% CI: 90.3–98.1%] for AZtrauma and 93.8% [95% CI: 92.2–95.3%] for AZchest.

More specifically, diagnostic consistency was maintained across all variables.

**Figure 1:**
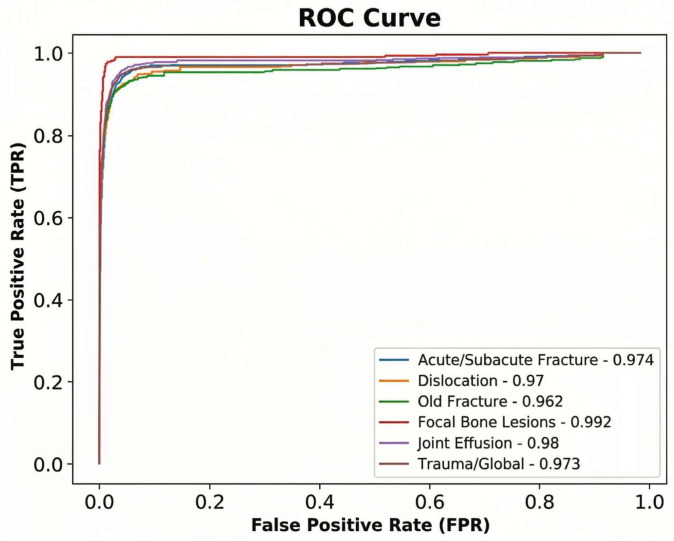
Receiver operating characteristic (ROC) curves of AZtrauma (per finding & global). No specific threshold or confidence intervals are shown.

For AZtrauma, AUC values remained highly consistent across age groups (96.7–97.7%) and sex (97.0–97.6%). Receiver operating characteristic (ROC) curves of AZtrauma (per finding & global) are shown in Figure 1.

**Figure 2:**
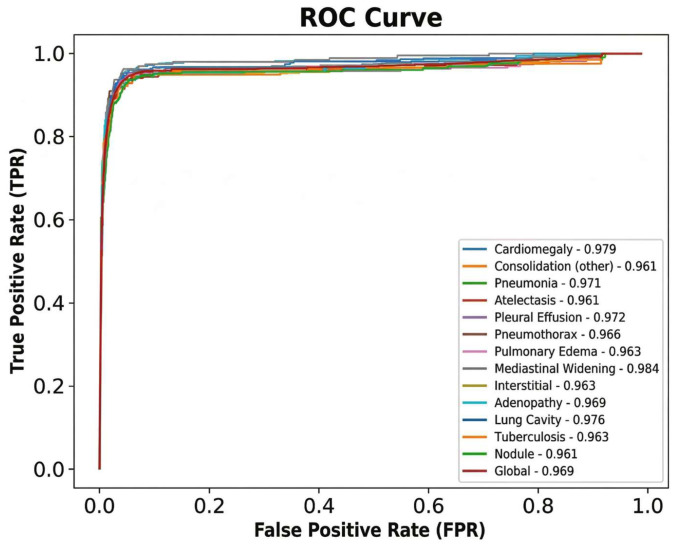
Receiver operating characteristic (ROC) curves of AZchest (per finding & global). No specific threshold or confidence intervals are shown.

Similarly, AZchest demonstrated stable discriminative power across demographics, with AUCs ranging from 0.9580 to 0.9733. Receiver operating characteristic (ROC) curves of AZchest (per finding & global) are shown in Figure 2.

Geographic analysis across 20 countries showed that AUC consistently exceeded 94.0% for AZtrauma and 93.8% for AZchest. Sensitivity remained high for both verticals, ranging from 91.8% [95% CI: 82.2–96.5%] to 98.7% [95% CI: 97.0–99.5%] for AZtrauma, and from 91.6% [95% CI: 88.6–93.8%] to 98.2% [95% CI: 94.7–99.4%] for AZchest.

Performance across all body regions showed minimal deviation, with a narrow AUC range (94.4–98.2%) for AZtrauma.

Detailed subgroup metrics are summarized in Table 4 for AZtrauma and Table 5 for AZchest.

## 4. Discussion

### 4.1. Global Resilience and Algorithmic Stability

The stability and consistency of the Rayvolve^®^ AI suite’s performance across 20 countries and diverse demographic strata, as evidenced by overlapping confidence intervals, constitute a significant milestone in validating deep learning tools for global clinical application [8,28]. Unlike many AI models that exhibit “site-dependency” or experience performance deterioration when exposed to out-of-distribution data, often referred to as “algorithmic drift”, the Rayvolve^®^ AI suite maintained a mean AUC of 97.5% for trauma and 96.7% for chest findings regardless of the acquisition setting. This consistency is particularly noteworthy in the pediatric (ages 0–18 years) and geriatric (ages over 60 years) subgroups. In the pediatric population, the challenges associated with skeletal maturation, including open growth plates and evolving ossification centers, often confound both human interpretation and standard automated algorithms [18,21]. Similarly, the geriatric demographic frequently manifests degenerative changes and osteopenia, which may obscure acute pathological findings. The minimal variance observed between disparate geographic regions suggests that the underlying ensemble architecture, which combines various models such as HRNet and PVT, is resilient to discrepancies in radiographic equipment, including variations in detector sensitivities from different manufacturers, exposure parameters, and anatomical diversity [11,12]. The robustness of our results is best contextualized through independent evaluations of the same system. In this regard, our findings are highly consistent with a recent study by Cohen et al. (2026) [35], which reported comparable performance metrics, including an AUC of 97.8% (95% CI: 97.6–97.9) for AZchest and 98.3% (95% CI: 98.2–98.4) for AZtrauma. This strong concordance across distinct large-scale cohorts further reinforces the algorithmic stability and clinical viability of the suite.

Additionally, according to the Landis and Koch criteria, a Cohen’s Kappa score of 0.83 indicates an “almost perfect agreement”. This aligns with our overall discordance rate of 4.77% (agreement of 95.23%), confirming the high reliability of the initial annotations prior to adjudication.

### 4.2. From Point Solutions to Unified Diagnostic Horizons

To our knowledge, the present study constitutes the first comprehensive, international evaluation of a unified AI suite that addresses eighteen distinct findings within a singular integrated workflow. Traditionally, the AI market for radiology has been dominated by the prevalence of point solutions, narrow algorithms that target specific body regions and/or pathologies. This fragmentation creates a significant “integration tax” for healthcare IT departments and complicates the radiologist’s workflow with the presence of multiple disparate interfaces [15].

A key observation of this research is the near-total parity in performance between the newly expanded diagnostic scope and historically validated findings. While established tasks like acute fracture detection have undergone years of iterative optimization (achieving AUCs up to 98.0%) [19,20], the new findings within the primary objective, such as mediastinal widening or focal bone lesions, demonstrated equivalent, and in some cases superior, discriminative power (AUCs up to 99.2%). This absence of a “performance gap” suggests that deep learning architectures have reached a level of maturity allowing for the rapid scaling of clinical scope without compromising diagnostic integrity [28].

Future iterations of the suite will expand into opportunistic screening (like AI-based Abdominal Aortic Calcification (AAC) detection), which efficiently automates risk stratification compared to current manual diagnostic methods.

### 4.3. Clinical Significance in MSK

The clinical utility of the expanded MSK scope is best illustrated by the algorithm’s performance on focal bone lesions (AUC = 99.2%). This suggests an strong ability to differentiate pathological bone density alterations, such as lytic or sclerotic metastases, from normal anatomical variations or benign islands, a task traditionally requiring high-level radiologic expertise [36]. By identifying such incidental findings during routine trauma evaluations, the system potentially reduces the satisfaction of search (SOS) bias. SOS is a well-documented cognitive error where the discovery of an obvious primary finding (e.g., a distal radius fracture) leads the clinician to prematurely terminate the search, overlooking secondary but potentially more life-threatening abnormalities [37]. In this context, the AI is designed to act as a persistent second pair of eyes, facilitating the early detection of occult malignancies or chronic infections that might otherwise remain undetected until a later clinical stage [23,24].

However, the analysis also highlighted the inherent biological challenges of bone remodeling; old fractures exhibited the lowest sensitivity (94.5%), likely reflecting the subtle nature of cortical remodeling and the variable appearance of callus formation compared to the sharp lucency of acute disruptions [4,7]. Conversely, the high sensitivity for joint effusion (97.8%) is a vital clinical metric, as effusion is often the only indirect sign of an intra-articular occult fracture in pediatric or emergency settings [9].

Comparatively, the diagnostic accuracy for acute/subacute fractures in this study (AUC = 97.4%) aligns with and reinforces results from previous Rayvolve^®^-specific validations, notably the AUCs reaching up to 95% reported by Dupuis et al. [18], Fu et al. [19], and Sadegi et al. [21] across diverse age groups. In a broader context, our results demonstrate superior performance compared to general MSK-AI literature; for instance, the systematic meta-analysis by Kuo et al. [7] reported a pooled AUC of 91% to 94% for fracture detection across various AI architectures. The higher performance observed in the current study, achieved on an international dataset, underscores the maturity of the ensemble-based approach in handling real-world radiographic variability.

### 4.4. Thoracic Performance and the Safety Net Paradigm

Within the thoracic domain, the high performance in detecting tuberculosis-related signs (lung cavities, hilar adenopathy, and interstitial patterns) underscores the system’s potential for global health impact, particularly in regions with limited access to specialized radiologists [25,27]. While pneumothorax exhibited the lowest relative sensitivity (94.5%), this reflects the known technical difficulty of identifying small, non-displaced apical lesions on standard radiographs [14].

Crucially, the consistently high NPV across all thoracic and MSK findings (exceeding 99.1%) validates the suite’s role as a reliable clinical rule-out tool. In high-pressure environments like Emergency Departments, a high NPV allows for the confident identification of normal X-rays, potentially accelerating patient discharge and reducing cognitive load on physicians by allowing them to focus their attention on positive, complex cases [38]. Furthermore, understanding the acceptable clinical trade-offs is essential. To maintain this high NPV and ensure the system functions as a rigorous safety net, the operational thresholds inherently accept a higher rate of False Positives. It should be noted that the relatively low PPVs observed for specific findings (e.g., 8.3% for TB and 10.6% for lung cavities) are a direct consequence of the low prevalence of these conditions in our cohort, as PPV is inherently prevalence-dependent. Under these conditions, achieving a high NPV, which is the clinical priority for a screening tool, necessitates this trade-off. In acute triage scenarios, this is a clinically acceptable compromise: over-flagging a potential subtle consolidation (a False Positive requiring brief human verification) is vastly preferable to missing a critical, life-threatening abnormality like a pneumothorax (a False Negative). Therefore, the primary beneficiaries of this system are frontline, non-specialized clinicians who require high-sensitivity automated oversight to prevent critical diagnostic omissions.

AZchest results (mean AUC = 96.7%) are highly consistent with the prior performance evaluation of the Rayvolve^®^ AI suite by Bettinger et al. [20], which observed AUCs exceeding 95% for six thoracic abnormalities included in our study. Furthermore, when compared to independent state-of-the-art chest AI models, our findings for specific pathologies like tuberculosis (AUC = 96.4%) and pulmonary nodules (AUC = 96.1%) align closely with the performance ranges reported in external literature. For example, meta-analyses by Qin et al. [25] for tuberculosis detection and large-scale studies by Hwang et al. [14] and Seah et al. [13] for emergency chest interpretation typically report AUC values between 90% and 95%. This comparison suggests that a unified, multi-label diagnostic approach can achieve highly competitive diagnostic reliability comparable to dedicated single-domain solutions. Nevertheless, performance metrics for highly localized pathologies must be contextualized geographically, specifically for tuberculosis. The majority of positive cases in our dataset originated from India. While this accurately reflects the disease’s real-world global epidemiological situation [39], it introduces a potential limitation. The algorithm’s high performance for tuberculosis detection may be partially optimized for the demographic characteristics, clinical phenotypes, and radiographic protocols prevalent in that high-incidence region. Consequently, ensuring its generalizability to lower-incidence populations or different genetic demographics will require further targeted cross-regional validation.

### 4.5. Challenges in the Analysis and Current Missed Findings

Beyond quantitative metrics, a qualitative analysis of FNs highlights specific physiological and technical challenges. In musculoskeletal imaging, FNs were primarily associated with very small lytic focal bone lesions (<5 mm) or those located in anatomically complex regions with significant cortical overlap, such as the sacrum or pelvic ring. Additionally, overlapping temporal features occasionally led to subacute fractures being misclassified as old. In chest radiography, FNs for mediastinal widening and adenopathy frequently occurred in images with suboptimal inspiratory volume or significant patient rotation. Missed tuberculosis signs and small lung cavities (<1 cm) were typically linked to subtle, low-density apical scarring partially obscured by the clavicles or upper ribs. Finally, early-stage consolidations with low attenuation, as well as fine interstitial patterns in patients with large body habitus or poor exposure, were occasionally missed due to a diminished signal-to-noise ratio in the lung parenchyma.

**Figure 3:**
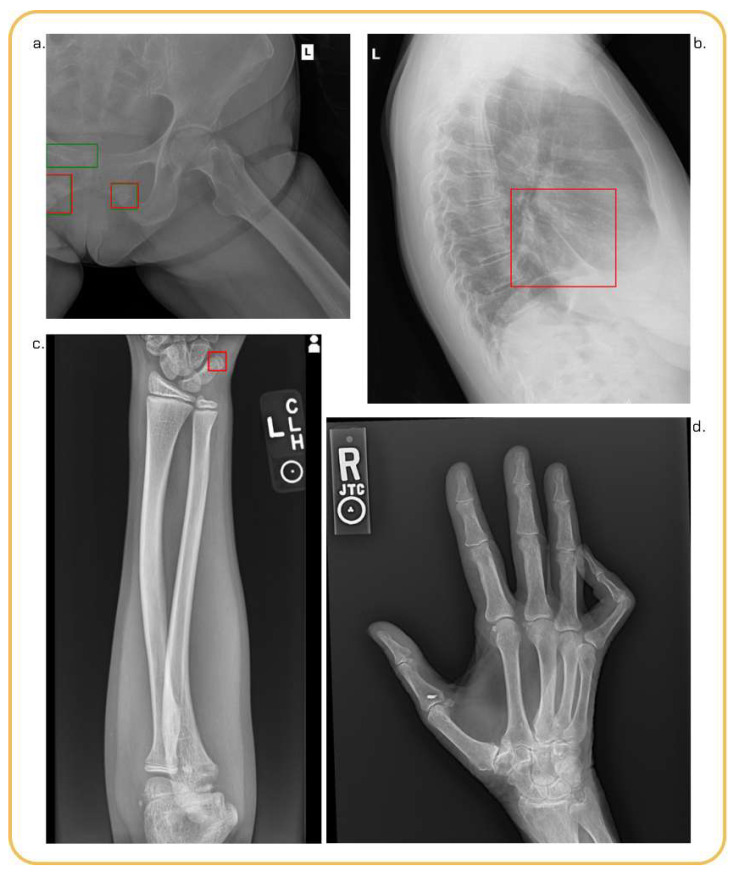
Examples of standalone performance of the Rayvolve^®^ AI suite. Green and red boxes respectively represent GT and the AI model predictions. (**a**) AZtrauma correctly identified 2 fractures (TP) and missed the ilio pubic ramus fracture (FN). (**b**) AZchest incorrectly detected pleural effusion on the lateral view of lung (FP). (**c**) AZtrauma incorrectly detected a fracture on the right hand (FP). (**d**) AZtrauma correctly identified a normal radiography (TN).

Examples of TP, TN, FP, and FN studies are shown in Figure 3.

### 4.6. Strengths and Limitations

The principal strength of this research is its innovative multicenter design, utilizing a large, sequestered, and independent dataset to ensure external validity. This external validation on geographically diverse data is a reliable method to mitigate shortcut learning and ensure that the model generalizes across varying radiographic equipment and patient morphologies. By incorporating real-world variability, including images with suboptimal positioning, artifacts, and varying resolutions, the findings effectively mirror routine clinical practice and support its inherent complexity [28].

However, several limitations persist. The retrospective nature of the study, while essential for a comprehensive evaluation of eighteen findings, may introduce selection bias and remains a lower level of evidence compared to prospective trials. The AI suite was evaluated in a standalone configuration without a concurrent comparison to human diagnostic performance. Consequently, the impact of the system on reducing specific cognitive biases or improving overall diagnostic accuracy in a real-world setting remains to be empirically demonstrated. Future multi-reader, multi-case (MRMC) studies are expected to formally benchmark the AI against expert radiologists and quantify its definitive contribution to clinical decision-making. A secondary limitation is potential label noise: despite a rigorous double-reading protocol, our GT is radiographically defined rather than pathologically proven. Blinding readers to collateral 3D imaging and follow-up data leaves the reference standard susceptible to the inherent visual limitations of 2D radiography. This reliance on a consensus of expert readers, rather than histopathological or cross-sectional confirmation (e.g., CT/MRI), may introduce a systematic bias. In particular, for complex findings like focal bone lesions or tuberculosis. Moreover, while our cohort is exceptionally large (*n* = 21,581), its real-world design results in very low prevalence for certain pathologies. High-priority findings like Tuberculosis (prevalence = 1.2%) and Lung Cavities (prevalence = 1.5%) have small positive sample sizes, potentially limiting statistical power. This highlights the challenge of hidden stratification and the need for highly specialized datasets to ensure equitable performance across the clinical long tail of rare signs and multi-systemic syndromes [22]. Additionally, the absence of skull and facial bone imaging limits complete trauma assessments.

Furthermore, the retrospective, multi-national data acquisition precluded full adherence to STARD-AI guidelines (e.g., reconstructing a comprehensive participant flow diagram). This reflects a broader challenge: the lack of strictly standardized validation protocols for multi-label AI, which complicates direct benchmarking.

Lastly, moving beyond standalone metrics, future research must employ prospective, multi-center randomized controlled trials (RCTs) to evaluate ‘human-in-the-loop’ synergy. It is imperative to quantify how this suite impacts definitive endpoints, including diagnostic turnaround time, ‘satisfaction of search’ errors, cost-effectiveness, and patient outcomes.

## 5. Conclusions

In conclusion, this large-scale international evaluation confirms the validation of the Rayvolve^®^ AI suite across an extensive range of diagnostic findings, encompassing eighteen distinct findings. The suite demonstrates high standalone performance within an expanded clinical domain, achieving accuracy levels comparable to historically optimized standards. This research illustrates the rapid advancement and scalability of deep learning models in medical imaging. It highlights a shift from narrow, task-specific applications to a more integrated, comprehensive diagnostic system.

However, these findings should be interpreted within the context of retrospective data. It is recognized that conventional radiography encompasses a vast spectrum of additional rare pathologies that remain unaddressed. Future development must focus on the iterative expansion of algorithmic libraries to capture the remaining breadth of the clinical long tail. Prospective, real-world trials are now warranted to quantify the definitive impact of this expanded AI diagnostic horizon on reducing diagnostic delay, healthcare costs, and overall patient morbidity in global clinical practice.

## Figures and Tables

**Table 1:** Descriptive Statistics of the Study Population per case.

Population	Subgroup	Total	AZtrauma	AZchest
**Age**	0–18 years old	5309	2520	2653
	19–60 years old	12,193	6423	5864
	>60 years old	4079	2182	1938
**Sex**	Male	10,769	5634	5193
	Female	10,812	5491	5263
**Country**	Argentina	553	189	364
	Australia	466	192	274
	Belgium	623	277	346
	Brazil	541	183	358
	Bulgaria	469	128	340
	Canada	473	386	87
	Estonia	615	160	455
	France	1244	1028	216
	Germany	1190	950	240
	India	1268	179	1089
	Israël	565	276	290
	Italy	953	226	727
	Morocco	1029	474	555
	Poland	858	634	224
	Portugal	1624	1175	449
	Romania	678	432	247
	Spain	2461	1244	1217
	Switzerland	1585	765	819
	UK	2078	416	1662
	USA	2309	1810	498
**Body Region**	Ankle	892	892	NA
	Clavicle	333	333	NA
	Chest	10,456	NA	10,456
	Elbow	802	802	NA
	Femur	401	401	NA
	Foot	499	499	NA
	Forearm	523	523	NA
	Hand	852	852	NA
	Hip/Pelvis	1034	1034	NA
	Humerus	106	106	NA
	Knee	807	807	NA
	Ribs	384	384	NA
	Shoulder	948	948	NA
	Tibia/Fibula	1309	1309	NA
	Wrist	1333	1333	NA
	Cervical Spine	195	195	NA
	Thoracic Spine	350	350	NA
	Lumbar Spine	357	357	NA

**Table 2:** Performance metrics for primary objective pathologies (Expanded Diagnostic Scope) per case.

Pathology (*n* = Prevalence)	AUC ^1^ (95% CI ^2^)	Se ^3^ (95% CI)	Sp ^4^ (95% CI)	PPV ^5^ (95% CI)	NPV ^6^ (95% CI)
**Hilar/Mediastinal adenopathy (2.3%)**	96.9% [95.5–98.1]	95.5% [92.1–97.5]	87.5% [86.8–88.1]	15.3% [13.6–17.2]	99.9% [99.8–99.9]
**Lung Cavity** **(1.5%)**	97.6% [96.0–98.9]	96.8% [92.8–98.6]	87.5% [86.9–88.1]	10.6% [9.1–12.3]	99.9% [99.9–100.0]
**Tuberculosis** **(1.2%)**	96.4% [93.9–98.4]	95.2% [90.0–97.8]	87.2% [86.5–87.8]	8.3% [7.0–9.8]	99.9% [99.9–100.0]
**Focal Bone Lesions (2.4%)**	99.2% [98.5–99.7]	98.9% [96.8–99.6]	96.1% [95.8–96.5]	38.6% [35.0–42.3]	100.0% [99.9–100.0]
**Interstitial Pattern** **(3.7%)**	96.3% [94.9–97.5]	95.6% [93.1–97.2]	87.7% [87.1–88.3]	23.0% [21.0–25.1]	99.8% [99.7–99.9]
**Mediastinal Widening (1.9%)**	98.4% [97.5–99.2]	97.5% [94.2–98.9]	86.6% [85.9–87.2]	12.2% [10.7–14.0]	99.9% [99.9–100.0]
**Pneumonia** **(5.8%)**	97.1% [96.1–97.9]	96.1% [94.2–97.3]	88.2% [87.5–88.8]	33.4% [31.3–35.7]	99.7% [99.6–99.8]
**Atelectasis** **(6.8%)**	96.1% [95.1–97.0]	95.1% [93.3–96.5]	87.5% [86.8–88.2]	35.8% [33.7–38.0]	99.6% [99.4–99.7]
**Old Fracture** **(8.4%)**	96.2% [95.3–97.0]	94.5% [92.8–95.8]	88.1% [87.4–88.7]	42.2% [40.1–44.3]	99.4% [99.2–99.6]

^1^ AUC: Area Under the Curve (AUC) of the Receiver Operating Characteristic (ROC). ^2^ CI: Confidence Interval. ^3^ Se: Sensitivity. ^4^ Sp: Specificity. ^5^ PPV: Positive Predictive Value. ^6^ NPV: Negative Predictive Value.

**Table 3:** Performance metrics for secondary objective pathologies (Previously Validated Findings) per case.

Pathology (*n* = Prevalence)	AUC ^1^ (95% CI ^2^)	Se ^3^ (95% CI)	Sp ^4^ (95% CI)	PPV ^5^ (95% CI)	NPV ^6^ (95% CI)
**Cardiomegaly** **(7.8%)**	97.9% [97.3–98.5]	97.8% [96.5–98.6]	84.6% [83.9–85.3]	34.8% [32.9–36.8]	99.8% [99.7–99.9]
**Consolidation** **(3.4%)**	96.1% [94.7–97.4]	95.2% [92.4–97.0]	85.8% [85.1–86.5]	18.9% [17.1–20.8]	99.8% [99.7–99.9]
**Pneumothorax** **(2.4%)**	96.6% [95.1–98.0]	94.5% [91.0–96.7]	89.4% [88.8–90.0]	18.2% [16.2–20.3]	99.9% [99.7–99.9]
**Pulmonary Nodule (3.8%)**	96.1% [94.8–97.3]	95.5% [93.0–97.1]	85.0% [84.3–85.7]	20.1% [18.4–22.0]	99.8% [99.7–99.9]
**Pleural Effusion** **(7.2%)**	97.2% [96.5–97.9]	96.7% [95.1–97.7]	86.5% [85.8–87.2]	35.7% [33.6–37.8]	99.7% [99.6–99.8]
**Pulmonary Edema** **(3.8%)**	96.3% [94.9–97.6]	95.7% [93.3–97.3]	88.5% [87.9–89.1]	24.8% [22.7–27.0]	99.8% [99.7–99.9]
**Acute/Subacute Fracture** **(20.4%)**	97.4% [97.0–97.9]	97.0% [96.3–97.7]	86.8% [86.1–87.5]	65.3% [63.7–66.9]	99.1% [98.9–99.3]
**Dislocation** **(4.5%)**	97.0% [95.9–97.9]	95.8% [93.6–97.2]	85.2% [84.5–85.9]	23.2% [21.5–25.1]	99.8% [99.6–99.8]
**Joint Effusion** **(5.7%)**	98.0% [97.2–98.7]	97.8% [96.4–98.7]	85.7% [85.0–86.4]	29.4% [27.5–31.4]	99.8% [99.7–99.9]

^1^ AUC: Area Under the Curve (AUC) of the Receiver Operating Characteristic (ROC). ^2^ CI: Confidence Interval. ^3^ Se: Sensitivity. ^4^ Sp: Specificity. ^5^ PPV: Positive Predictive Value. ^6^ NPV: Negative Predictive Value.

**Table 4:** AZtrauma Subgroup Analysis per case.

AZtrauma Subgroup (*n* = Prevalence)		AUC ^1^ (95% CI ^4^)	Se ^2^ (95% CI)	Sp ^3^ (95% CI)
**Age**	0–18 years old (7.5%)	96.7% [96.0–97.4]	95.8% [94.3–96.9]	86.1% [85.5–86.8]
	19–60 years old (7.7%)	97.5% [97.0–97.8]	96.8% [96.0–97.4]	90.2% [89.9–90.6]
	>60 years old (11.1%)	97.7% [97.1–98.2]	96.9% [95.7–97.7]	86.0% [85.3–86.7]
**Sex**	Male (8.4%)	97.0% [96.5–97.5]	96.3% [95.4–97.0]	88.6% [88.2–89.0]
	Female (8.1%)	97.6% [97.2–98.0]	97.0% [96.2–97.6]	88.4% [88.0–88.8]
**Country**	Argentina (8.0%)	97.5% [94.9–99.5]	97.4% [90.9–99.3]	85.9% [83.4–88.0]
	Australia (8.1%)	95.1% [91.2–98.3]	94.9% [87.5–98.0]	85.7% [83.3–87.9]
	Belgium (8.4%)	94.0% [90.9–96.9]	92.2% [85.9–95.9]	86.4% [84.4–88.2]
	Brazil (7.9%)	95.6% [92.1–98.6]	94.4% [86.6–97.8]	86.6% [84.1–88.7]
	Bulgaria (8.7%)	95.2% [91.2–98.5]	92.9% [83.0–97.2]	86.0% [83.0–88.6]
	Canada (8.6%)	95.9% [93.7–97.8]	94.6% [90.0–97.1]	85.8% [84.1–87.3]
	Estonia (8.1%)	96.3% [92.9–99.0]	95.4% [87.3–98.4]	86.6% [84.0–88.9]
	France (8.7%)	96.4% [95.2–97.4]	94.6% [92.1–96.4]	90.3% [89.5–91.1]
	Germany (8.2%)	98.5% [97.8–99.2]	98.7% [97.0–99.5]	86.6% [85.5–87.6]
	India (8.7%)	97.0% [94.4–99.1]	93.6% [85.9–97.2]	92.0% [90.0–93.7]
	Israël (8.6%)	95.9% [93.1–98.3]	95.8% [90.5–98.2]	86.0% [83.9–87.8]
	Italy (8.5%)	96.2% [93.7–98.4]	94.8% [88.4–97.8]	86.3% [84.1–88.2]
	Morocco (8.1%)	97.6% [96.1–98.9]	97.4% [94.1–98.9]	86.3% [84.8–87.7]
	Poland (8.1%)	97.4% [96.2–98.6]	96.5% [93.5–98.2]	87.9% [86.6–89.0]
	Portugal (8.2%)	98.7% [98.1–99.3]	98.3% [96.8–99.2]	95.4% [94.8–96.0]
	Romania (8.5%)	95.8% [93.8–97.6]	94.0% [89.6–96.6]	86.2% [84.6–87.7]
	Spain (7.8%)	97.7% [96.9–98.4]	97.3% [95.5–98.4]	86.1% [85.1–86.9]
	Switzerland (8.5%)	97.2% [96.0–98.4]	96.6% [94.1–98.1]	86.0% [84.8–87.1]
	United Kingdom (8.2%)	97.5% [96.1–98.7]	97.1% [93.3–98.7]	85.7% [84.1–87.2]
	United States (8.3%)	98.2% [97.5–98.8]	97.9% [96.6–98.7]	91.0% [90.3–91.6]
**Body region**	Ankle (8.3%)	98.0% [97.0–98.9]	97.6% [95.5–98.7]	92.6% [91.8–93.4]
	Clavicle (9.3%)	95.3% [93.0–97.3]	92.9% [87.7–96.0]	83.9% [82.0–85.7]
	Elbow (6.0%)	98.2% [97.3–99.1]	97.9% [95.2–99.1]	85.4% [84.2–86.5]
	Femur (9.9%)	94.4% [92.0–96.6]	92.9% [88.5–95.7]	91.1% [89.7–92.3]
	Foot (8.8%)	98.1% [97.3–98.8]	97.4% [95.8–98.4]	92.8% [92.2–93.5]
	Forearm (12.1%)	96.2% [94.8–97.5]	94.4% [91.2–96.5]	88.9% [87.5–90.1]
	Hand (9.0%)	98.2% [97.5–98.9]	97.5% [95.9–98.5]	94.4% [93.8–95.0]
	Hip/Pelvis (6.0%)	98.2% [97.3–99.0]	97.7% [95.4–98.9]	87.4% [86.5–88.3]
	Humerus (13.7%)	97.9% [96.3–98.4]	96.6% [94.2–98.1]	86.7% [85.2–88.0]
	Knee (7.2%)	98.0% [97.2–98.8]	98.0% [95.8–99.1]	83.8% [82.6–84.9]
	Ribs (11.5%)	94.4% [90.3–98.1]	91.8% [82.2–96.5]	90.0% [86.9–92.4]
	Shoulder (8.2%)	97.0% [95.7–98.1]	96.1% [93.4–97.7]	89.4% [88.4–90.3]
	Tibia/Fibula (11.0%)	98.0% [96.6–99.1]	97.6% [94.6–99.0]	91.1% [89.6–92.3]
	Wrist (6.4%)	97.8% [96.7–98.8]	97.4% [94.9–98.7]	82.6% [81.4–83.7]
	Cervical Spine (7.8%)	96.5% [93.3–99.1]	94.7% [87.2–97.9]	84.2% [81.6–86.4]
	Thoracic Spine (5.9%)	96.2% [93.9–98.3]	95.2% [89.1–97.9]	81.5% [79.6–83.3]
	Lumbar Spine (5.9%)	96.2% [93.6–98.4]	94.3% [88.1–97.4]	85.9% [84.2–87.5]

^1^ AUC: Area Under the Curve (AUC) of the Receiver Operating Characteristic (ROC). ^2^ Se: Sensitivity. ^3^ Sp: Specificity, ^4^ CI: Confidence Interval.

**Table 5:** AZchest Subgroup Analysis per case.

AZchest Subgroup (*n* = Prevalence)		AUC ^1^ (95% CI ^4^)	Se ^2^ (95% CI)	Sp ^3^ (95% CI)
**Age**	0–18 years old (4.0%)	97.3% [96.7–97.8]	96.4% [95.3–97.3]	86.6% [86.2–87.0]
	19–60 years old (3.7%)	97.3% [96.9–97.7]	96.5% [95.8–97.1]	87.8% [87.6–88.0]
	>60 years old (4.8%)	95.8% [95.0–96.5]	94.7% [93.3–95.8]	85.5% [85.1–86.0]
**Sex**	Male (3.8%)	97.3% [96.9–97.7]	96.9% [96.2–97.5]	86.4% [86.1–86.7]
	Female (4.1%)	96.5% [96.0–96.9]	95.3% [94.5–96.0]	87.8% [87.5–88.0]
**Country**	Argentina (3.9%)	97.9% [96.5–99.2]	97.8% [94.6–99.2]	85.8% [84.8–86.8]
	Australia (4.5%)	96.2% [94.0–98.0]	94.3% [89.6–97.0]	87.2% [86.0–88.3]
	Belgium (3.9%)	97.9% [96.7–99.0]	97.7% [94.3–99.1]	86.4% [85.4–87.4]
	Brazil (3.5%)	98.1% [96.9–99.2]	98.2% [94.7–99.4]	85.6% [84.5–86.6]
	Bulgaria (3.0%)	98.1% [96.5–99.3]	97.7% [93.5–99.2]	85.2% [84.1–86.3]
	Canada (3.5%)	97.2% [93.0–99.7]	97.4% [86.8–99.6]	86.3% [84.1–88.2]
	Estonia (4.7%)	95.4% [93.5–97.0]	93.5% [89.9–95.8]	87.2% [86.3–88.1]
	France (3.9%)	97.6% [95.7–99.1]	96.4% [91.0–98.6]	86.4% [85.1–87.7]
	Germany (4.6%)	94.9% [92.3–97.2]	93.1% [87.7–96.2]	86.5% [85.2–87.7]
	India (4.3%)	97.1% [96.1–97.9]	96.2% [94.4–97.5]	92.1% [91.7–92.6]
	Israël (3.0%)	98.1% [96.3–99.5]	98.2% [93.8–99.5]	85.8% [84.6–86.9]
	Italy (4.6%)	93.8% [92.2–95.3]	91.6% [88.6–93.8]	87.3% [86.6–88.0]
	Morocco (5.0%)	95.8% [94.3–97.1]	93.6% [90.5–95.7]	87.6% [86.8–88.3]
	Poland (4.6%)	96.9% [95.0–98.5]	95.5% [90.6–97.9]	87.0% [85.7–88.2]
	Portugal (3.4%)	97.9% [96.7–99.0]	98.0% [95.0–99.2]	86.3% [85.3–87.1]
	Romania (4.1%)	94.9% [92.3–97.3]	92.4% [86.6–95.8]	87.0% [85.7–88.1]
	Spain (3.1%)	98.2% [97.4–98.8]	98.0% [96.3–98.9]	86.7% [86.2–87.3]
	Switzerland (4.0%)	97.9% [97.0–98.7]	97.9% [96.0–98.9]	86.1% [85.5–86.8]
	United Kingdom (4.1%)	97.5% [96.9–98.2]	97.0% [95.7–97.9]	86.3% [85.9–86.8]
	United States (3.3%)	97.6% [96.1–98.8]	97.7% [94.7–99.0]	86.0% [85.1–86.8]

^1^ AUC: Area Under the Curve (AUC) of the Receiver Operating Characteristic (ROC). ^2^ Se: Sensitivity. ^3^ Sp: Specificity, ^4^ CI: Confidence Interval. Using the spatial matching approach with the IoU, the overall Cohen’s Kappa between the two initial readers was 0.83.

## Data Availability

The datasets analyzed during the current study are not publicly available due to the proprietary nature of the commercial medical data from the 52 participating centers but may be available from the corresponding author on reasonable request.

## Abbreviations

The following abbreviations are used in this manuscript:

AAC	Abdominal Aortic Calcification
AI	Artificial Intelligence
AP	Anteroposterior
AUC	Area Under the Curve
CI	Confidence Interval
CNIL	Commission on Informatics and Liberty
CNNs	Convolutional Neural Networks
CR	Computed Radiography
CT	Computerized Tomography scan
DICOM	Digital Imaging and Communications in Medicine
DL	Deep Learning
DR	Digital Radiography
FN	False Negative
FP	False Positive
GT	Ground Truth
HRNet	High-Resolution Networks
IoU	Intersection-over-Union
kVp	kiloVoltage
mAs	milliampere-seconds
MR	Méthodologie de Référence (see CNIL)
MRI	Magnetic Resonance Imaging
MSK	Musculoskeletal
NPV	Negative Predictive Value
PA	Posteroanterior
PPV	Positive Predictive Value
PVT	Pyramid Vision Transformer
RF	Radiographic Fluoroscopy
ROC	Receiver Operating Characteristic
STARD-AI	Standards for Reporting Diagnostic Accuracy AI
TN	True Negative
TP	True Positive
VGG	Visual Geometry Group
X-ray	Radiograph
